# Human mesenchymal stem cells treatment improved hepatic lesions and reversed gut microbiome disorder in non-alcoholic steatohepatitis

**DOI:** 10.18632/aging.103962

**Published:** 2020-11-08

**Authors:** Zhiyu Yang, Qiaoyun Xia, Di Lu, Han Yue, Jianmin Zhang, Yalong Li, Bingyong Zhang, Xiuling Li, Mingbo Cao

**Affiliations:** 1 Department of Gastroenterology, Henan Provincial People’s Hospital, People's Hospital of Zhengzhou University, School of Clinical Medicine, Henan University, Zhengzhou 450003, Henan, China; 2 Microbiology Laboratory, Henan Provincial People's Hospital, People's Hospital of Zhengzhou University, School of Clinical Medicine, Henan University, Zhengzhou 450003, Henan, China; 3 Stem Cell Research Center, Henan Key Laboratory of Stem Cell Differentiation and Modification Henan Provincial People’s Hospital, People’s Hospital of Zhengzhou University, School of Clinical Medicine, Henan University, Zhengzhou 450003, Henan, China

**Keywords:** non-alcoholic steatohepatitis, human mesenchymal stem cells, intestinal flora, methionine–choline-deficient diet, metabolome

## Abstract

Effective therapies for non-alcoholic steatohepatitis (NASH) are urgently needed. We investigated the effect of human mesenchymal stem cells (hMSCs) on the intestinal flora in NASH treatment. We isolated the hMSCs from the umbilical cords and divided male C57BL/6 mice into three groups, namely, chow, methionine–choline-deficient (MCD), and MCD+hMSCs. After collecting the feces and liver of the mice, we evaluated the histological changes in the liver and measured the inflammatory and fibrogenesis cytokines. Fecal microbiome and metabolome were analyzed using 16S rRNA gene sequencing analyses. The hMSCs treatment could alleviate hepatic steatosis, inflammation and fibrosis induced by MCD diet. It could also reverse the microbiome and metabolome disorders in the NASH model. Correlation analysis of the interaction among bacteria amplified the effects of the bacteria in host. In conclusion, hMSCs treatment could improve NASH-related lesions and reverse gut microbiome and metabolome disorder in NASH.

## INTRODUCTION

Non-alcoholic fatty liver disease (NAFLD) represents a spectrum of disorders ranging from simple steatosis to nonalcoholic steatohepatitis (NASH) characterized by inflammation with the potential progression to fibrosis and cirrhosis over time [1]; NAFLD has become the most common chronic liver diseases in the world [2]. No specific medicine exists for this disease. For patients with early NAFLD, diet and exercise are the main treatment options. With progressing liver lipotoxicity, cellular stress, and inflammation, one third of NAFLD patients progressed to NASH and fibrosis in the next 4-5 years [3]. Moreover, NASH is rapidly becoming an indicator for liver transplantation (LT) and has become the second leading etiology for LT in the United States [4, 5]. However, LT as a therapy is limited by the shortage of organs and the high rate of complications [6]. Therefore, an economical and effective therapeutic approach to prevent the progression of NAFLD to NASH is urgently needed. Recent studies have indicated that gut microbiota might play a critical role in the onset of NAFLD and the progression to NASH [1].

The liver obtains more than 70% of its blood from the gut through the portal vein, and the beneficial substances produced from the liver are absorbed by the gut, hence the term gut-liver axis [7]. The intestinal tract contains a large number of bacteria, which help the body absorb energy and nutrients. These bacteria can also secrete a variety of regulatory factors to affect the body. For example, some Gram-positive bacteria can produce butyrate, which could increase insulin sensitivity, decrease inflammatory cytokines, and upregulate lipid metabolism. Butyrate can also alleviate liver injury, fibrosis progression, and intestinal barrier dysfunction [8, 9]. However, some gut flora may produce harmful substances, such as lipopolysaccharide, which comprises the outer membrane of Gram-negative bacteria. The displacement and increase of lipopolysaccharide leads to the occurrence of inflammation, induces liver injury and fibrosis [10], and could lead to death in severe cases [11]. Intestinal mucosa is the first barrier that prevents bacteria and their metabolites from entering the body through the portal vein. Maintenance of the intestinal homeostasis and barrier integrity relies on the complex interaction between the host immune system and commensal microbiota [12].

Dysbiosis of gut microbiota and intestinal barrier dysfunction will increase bacterial translocation, and induce pro-inflammatory components and metabolites from bacteria into the liver. Ultimately, it may induce an acceleration in occurrence and development of NASH [7]. Both probiotics and microflora transplantation could significantly improve liver function and reduce liver inflammation of NASH [13, 14]. Meanwhile, accumulating clinical and animal studies have indicated that NAFLD is intimately associated with disruption of the balance between Firmicutes and Bacteroidetes [15, 16]. Recently, some researchers have studied the therapeutic effect of human umbilical cord-derived mesenchymal stem cells (hMSCs) on NASH and have found that hMSCs could alleviate hepatic functional injury, attenuate hepatic steatosis, and reduce hepatic lipid accumulation [17]. However, the effect of hMSCs on the intestinal flora of NASH remains unclear.

Mesenchymal stem cells (MSCs) are pluripotent stem cells with the functions of self-replication and multidirectional differentiation. Recent evidence suggests that MSCs play a significant role in some diseases [18, 19]. MSCs function as follows. Firstly, recruited MSCs could differentiate into functional cells to replace damaged cells. Secondly, MSCs could produce immunoregulatory factors that inhibit the progression of inflammation by affecting dendritic cells, B cells, T cells, and macrophages [20, 21]. For some diseases, such as cirrhosis of the liver, MSCs could produce a large amount of cytokines and growth factors, which stimulate angiogenesis, prevent apoptosis, block oxidation reactions, promote remodeling of the extra-cellular matrix, and induce the differentiation of tissue stem cells [20, 21]. Although litter MSCs that were injected into mice migrated to the liver and disappeared after 7days, they obviously contributed to liver fibrosis regression [22]. In various kinds of liver diseases, MSCs could ameliorate hepatic inflammation and fibrosis, as well as improve liver function and reduce ascites in patients. MSCs could also inhibit hepatocellular death and reverse fulminant hepatic failure [23, 24]. However, the effect of MSCs on the intestinal flora in the treatment of NASH is still unknown.

The gut-liver axis plays an extremely important role in regulating liver microenvironment and intestinal microecology [7]. The current study aimed to explore the correlation between liver microenvironment and intestinal microecology and to further clarify the mechanism underlying the use of MSCs as therapy for NASH. Our study might provide a new treatment for NASH.

## RESULTS

### Human umbilical cord MSCs were successfully isolated

**Figure 1:**
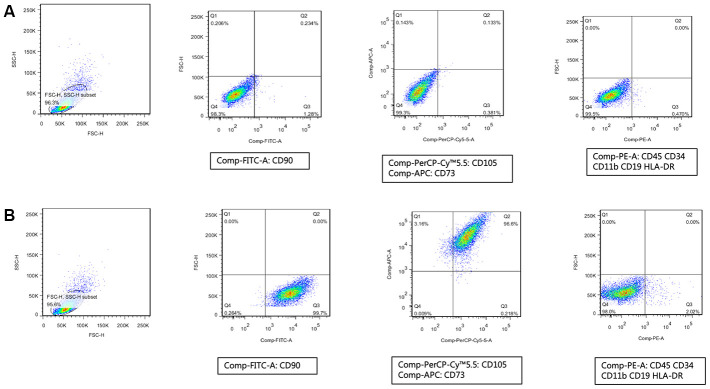
**Human umbilical cord mesenchymal stem cells were successfully isolated.** (**A**) Isotype antibodies were used as the control group of hMSCs. (**B**) Immunophenotypic features of hMSCs include being positive for CD90, CD105, and CD73 and negative for CD34, CD45, CD11b, CD19, and HLA-DR.

MSCs have specific surface molecular markers; they are positive for CD73, CD90, and CD105 and negative for CD34, CD45, CD11b, CD19, and HLA-DR [25, 26]. In our study, we first used flow cytometry to analyze and identify MSCs extracted from umbilical cord. As shown in Figure 1B, we first tested the positive molecular markers (CD90, CD105, and CD73) on the surface of MSCs, which were marked by FITC, PerCP-Cy™5.5, and APC, respectively. Subsequently, we tested the negative molecular markers (CD34, CD45, CD11b, CD19, and HLA-DR) on the surface of MSCs, which were marked by PE (Figure 1B). We used isotype antibodies as the control group (Figure 1A). When compared with the control group, the cells we used were verified as hMSCs.

### hMSCs alleviated hepatic steatosis, inflammation, and fibrosis induced by MCD diet

C57BL/6 mice were fed with MCD diet for 12 weeks to induce NASH-related lesions, and hMSCs were administered intravenously via tail injection at week 10. As control, the resuspending liquid of hMSCs were administrated intravenously in C57BL/6 mice fed with chow and MCD at week 10.

**Figure 2:**
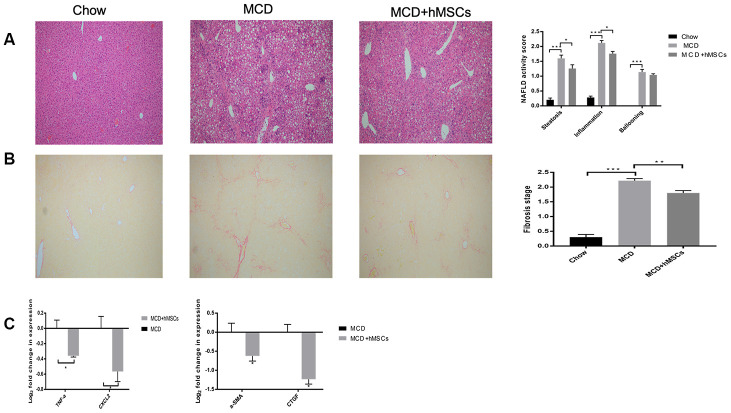
**Human mesenchymal stem cells alleviated hepatic steatosis, inflammation, and fibrosis induced by MCD diet.** (**A**) Representative images of HE-stained liver sections of the three groups. Steatosis, ballooning, and inflammation in the liver of the three groups were scored according to the NAFLD activity scoring system. (**B**) Representative images of Sirius Red-stained liver sections of the indicated groups. The fibrosis stages were evaluated according to the NAFLD activity score. (**C**) Relative mRNA levels of inflammatory cytokines (TNF-a and CXCL-2) and indicators of hepatic fibrosis (a-SMA and CTGF) from the MCD and MCD+hMSCs groups. * P < 0.05, ** P < 0.01, and *** P < 0.001.

As shown in Figure 2A, pathological sections of the mice liver were used for HE staining, and hepatic steatosis, inflammation, and ballooning were scored according to the NAFLD activity scoring system [27]. Compared with the chow group, MCD diet induced extensive macrovesicular fat accumulation along with infiltration of inflammatory cells, which were similar to the pathological changes of NASH in human patient. The administration of hMSCs significantly alleviated hepatic steatosis and inflammation induced by MCD diet compared with the MCD group. The mice liver sections were stained with Sirius red to evaluate the fibrosis of the liver, and the fibrosis stages were divided according to the NAFLD activity scoring system (Figure 2B). The MCD diet induced hepatic fibrosis compared with the chow diet, and the hMSCs treatment effectively alleviated liver fibrosis. To further clarify the alleviation of NASH-related lesions due to hMSCs therapy, we subsequently used PCR to analyze several inflammatory and fibrosis-related cytokines at the transcription level. As shown in Figure 2C, the inflammatory cytokines of TNF-a and CXCL-2 and the fibrogenesis cytokines of a-SMA and CTGF were all significantly downregulated in the MCD+hMSCs group compared with the MCD group.

### hMSCs improved the diversity of mouse flora

To further explore the mechanism by which stem cells improve NASH, we investigated the impact of hMSCs on the gut microbiome through the metagenomic sequencing of the 16S rRNA.

**Figure 3:**
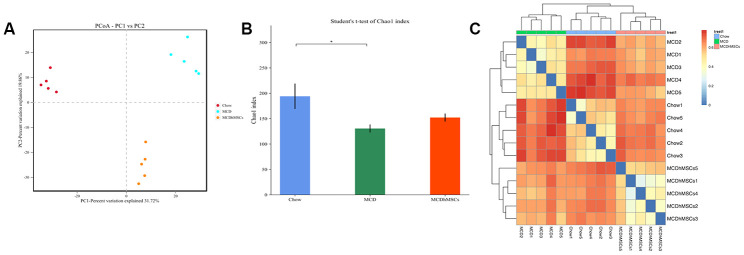
**Human mesenchymal stem cells improved the diversity of mouse flora.** (**A**) PCoA plot of the microbiota based on unweighted UniFrac metric. The three scatter plots represent the chow, MCD, and MCD+hMSCs groups. (**B**) The picture of Chao1 that represents alpha diversity of mouse flora indicated the difference among the three groups. * P < 0.05. (**C**) The binary Jaccard clustering health map represents integrity differences in the mice flora among the three groups.

First, we analyzed the principal components of intestinal flora among different groups, which was performed to calculate the beta-diversity values. As shown in Figure 3A, three groups, namely, the chow, MCD, and MCD+hMSCs groups, showed a clustering tendency in the first predictive principal component (X axis) and second predictive principal component (Y axis) in the unweighted UniFrac principal coordinate analysis (PCoA). Next, we conducted alpha diversity analysis (Chao1) of mouse flora, which represented the stability of intestinal flora. As shown in Figure 3B, the number of intestinal flora in MCD diet group was significantly lower than that in the chow group, and hMSCs treatment increased the number of intestinal flora despite that no statistical difference was achieved. Subsequently, we analyzed the binary Jaccard clustering health map to further observe the integrity differences in the mice flora among the three groups. As depicted in Figure 3C, the hMSCs treatment effectively alleviated the flora disorder induced by MCD diet.

### hMSCs reversed the microbiome disorder induced by MCD diet

The hMSCs treatment alleviated intestinal flora disorder induced by MCD diet. Next, to further explore the distribution difference of intestinal flora among the three groups, we studied flora diversity at the level of family, phylum, and species.

**Figure 4:**
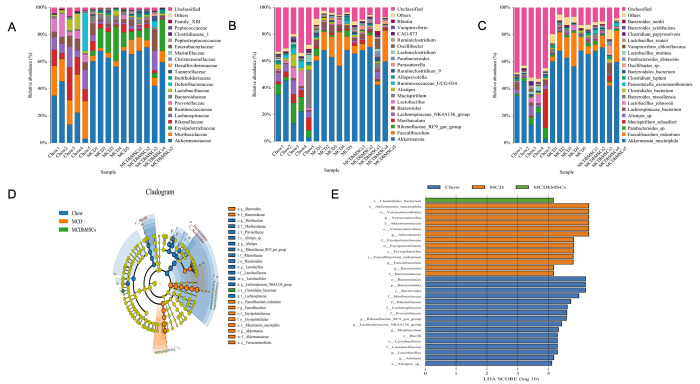
**Human mesenchymal stem cells reversed the microbiome disorder induced by MCD diet.** (**A**–**C**) The most abundant taxa at the family (**A**), genus (**B**), and species (**C**) levels. (**D**) LEfSe cladogram representing taxa enriched in the chow, MCD and MCD + hMSCs groups. Rings from inside out represented taxonomic levels from phylum to genus levels. Sizes of circles indicate the relative abundance of the taxa. (**E**) Discriminative biomarkers with an LDA score > 4.0.

As shown in Figure 4A, at the family level, the main intestinal flora of chow group were *Akkermansiaceae* and *Muribaculaceae*, which belong to *Verrucomicrobia* and *Bacteroidetes*, respectively, according to the phylogenetic tree. However, in the MCD diet group, *Akkermansiaceae* and *Erysipelotrichaceae* increased significantly compared with the chow group, accompanied with a decrease of *Muribaculaceae*. hMSCs treatment alleviated the flora disorder to a certain extent, but the effect was not remarkable. At the genus level, the hMSCs treatment significantly increased the quantity of *Bacteroides*, which decreased remarkably in MCD group (as shown in Figure 4B). Meanwhile, the flour of CAG-873, which belongs to *Muribaculaceae* [28], decreased definitely in the MCD group compared with the chow group, whereas the hMSCs treatment reversed this change of flora effectively. Similarly, hMSCs treatment restored the flora disorder of *Desulfovibrio* and *Odoribacter* caused by MCD diet (Figure 4B). At the species level, *Bacteroidales* bacteria community in the MCD group was significantly lower than that in the chow group, and hMSCs treatment increased the community remarkably (as shown in Figure 4C). Similarly, hMSCs restored the change in the *Bacteroides thetaiotaomicron* induced by MCD diet (Figure 4C). *Parabacteroides distasonis* showed no significant difference between MCD group and chow group, but its quantity was higher in the MCD+hMSCs group compared with the MCD group (Figure 4C).

To further identify the distinguishing phylotypes in the gut microbiota of the different groups, LEfSe analysis was performed. Compared with the chow group, *lactobacillusaceae*, *Rikenellaceae*, *Alistipes*, and *Muribaculaceae* decreased significantly, whereas *Akkermansiaceae*, *Erysipelotrichaceae*, *Faecalibaculum* and *Bacteroides* increased significantly in MCD group (as shown in Figure 4D). hMSCs treatment increased the numbers of Clostridia and alleviated MCD diet-induced changes in the microbiota flora (as shown in Figure 4E).

### hMSCs ameliorated disorders of metabolomic profiles induced by MCD induced diet

hMSCs could improve the intestinal flora disorder induced by MCD diet to some extent. To further elucidate the beneficial role of the altered microbial community, we studied the metabolic pathway and the interaction relationship of species among intestinal flora.

**Figure 5:**
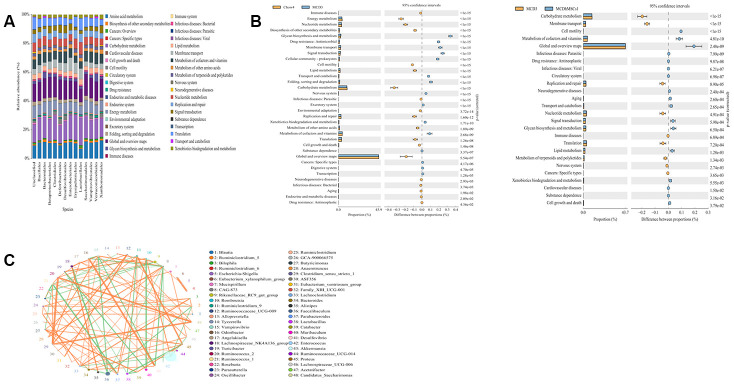
**Human mesenchymal stem cells ameliorated the disorders of metabolomic profiles induced by MCD diet.** (**A**) The expression levels of different species in various metabolic pathways. The x-coordinate represents species of the flora, and the y-coordinate represents the percentage of relative abundance of metabolic pathways. (**B**) Differences in the metabolic pathways between Chow4 and MCD3; MCD3 and MCD+hMSCs1 were showed in the picture. (**C**) The interaction among different bacterial communities was represented by Spearman’s rank correlation. Significant associations with P < 0.05 and r > 0.5 were shown. The circle represented species, and the circle size represented the average abundance of species. The line represented the correlation between the two species. The thickness of the line represented the strength of the correlation. The orange color of the line represented positive correlation, whereas green represented the negative correlation.

First, to measure the levels of individual functional distribution (the fecal metabolome) and the abundance of metabolic pathways in individual microbial species, we studied the KEGG metabolic pathways and divided the function of bacteria into six categories at the highest level, namely, metabolism, genetic information processing, human diseases, environmental information processing, cellular processes, and organizational systems. Furthermore, we performed more detailed divisions on the basis of these above mentioned categories. As shown in Figure 5A, we found that the metabolic pathways involved in each flora differed at the order level. For example, compared with the bacteria *Erysipelotrichaeae* and *Desulfovibrio*, *Lactobacillusaceae* and *Clostridiales* are more abundant in the lipid metabolism pathway.

Moreover, the difference of metabolic pathway was also obvious among the three groups of mice intestinal microflora (as shown in Figure 5B). Compared with the chow group, the MCD group had lower abundance in energy metabolism, nuclear metabolism, biosynthesis of secondary metabolism, carbohydrate metabolism, cell mobility, and lipid metabolism but showed a relatively increased expression in drug resistance and membrane transport. In the hMSCs treatment group, energy metabolism, carbohydrate metabolism, cell mobility, and lipid metabolism improved compared with the MCD group.

In addition, we found that intestinal flora can also play a role through the interaction among the flora. To clarify the correlation among species, we conducted Spearman rank correlation analysis according to the interaction of intestinal flora and built the correlation network with the data P < 0.05 and r > 0.5. As shown in Figure 5C, *Desulfovibrionales* had positive correlation with *Odoribacter* and negative correlation with *Anaerotruncus*. *Muribaculum* was negatively correlated with *Turicibacter*, *Desulfovibrio*, *Faecalibaculum*, and *Akkermansiaceae*.

## DISCUSSION

Studies showed that MSCs could alleviate the inflammation and fibrosis of NASH [18], but the effect on intestinal flora during this period was unclear. Our results showed that beyond reducing inflammatory molecules and alleviating liver fibrosis, hMSCs could also reverse the disorder of NASH mice flora induced by the MCD diet.

MSCs had been extensively explored for the capability of self-renewal and differentiation into various cell types and used as a therapeutic strategy for tissue regeneration and repair [19, 29, 30]. The source of MSCs was abundant, and the most widely studied sources of MSCs included bone marrow, adipose, muscle, peripheral blood, umbilical cord, placenta, fetal tissue, and amniotic fluid [31]. Among them, human umbilical cord-derived MSCs possess unique advantages because of their relative accessibility and abundance compared with other MSCs [32]. In our study, we used MSCs derived from human umbilical cord. Previous studies have confirmed that hMSCs have specific surface molecular markers, such as positive CD73, CD90, and CD105 and negative CD34, CD45, CD11b, CD19, and HLA-DR [25, 26]. hMSCs used in our study were confirmed by flow cytometry to have positive molecular markers (CD90, CD105, and CD73) and negative molecular markers (CD34, CD45, CD11b, CD19, and HLA-DR) on the surface of cells. Thus, the stem cells we used in our study were indeed hMSCs.

In our study, C57BL/6 mice were fed with MCD diet for 12 weeks to induce NASH-related lesions, and hMSCs were administrated intravenously via tail injection at week 10. Several reports have demonstrated that MSCs could reduce weight loss, steatosis, ballooning, and lobular inflammation in NASH induced by MCD diet [18, 21, 33]. In our current study, compared with the MCD group, hMSCs treatment significantly alleviated hepatic steatosis, inflammation, and liver fibrosis, which was consistent with the results of previous studies. Furthermore, we used PCR to analyze several inflammatory and fibrosis-related cytokines at the transcription level to observe the therapeutic effect of hMSCs therapy on NASH-related lesions. hMSCs treatment significantly downregulated the inflammatory cytokines of TNF-a and CXCL-2, which were significantly increased in NASH compared with the control group according to previous studies [34, 35]. hMSCs treatment also reduced the fibrogenesis cytokines of a-SMA and CTGF compared with the MCD group. Therefore, hMSCs may have potential as a therapy for NASH.

The mechanism of hMSCs involved in improving NASH-related lesions has not been clarified. As reported, the gut-liver axis, which represents the close relationship between the liver and the gut, might play a critical role in NAFLD onset and progression [1, 36]. To further explore the mechanism by which hMSCs alleviate NASH-related lesions, we investigated the impact of hMSCs on the gut microbiome using metagenomic sequencing of the 16S rRNA. Under the PCoA that represents the beta-diversity values, we found that the first and second predictive principal components were significantly different in all three groups, which stood for chow, MCD, and MCD+hMSCs groups. This results might prove the presence of differences among the three groups in the intestinal microenvironment. Many microbial communities exist in the human gut, and these communities promote the metabolism and digestion of the host. The diversity of intestinal flora is in a dynamic balance, and decreased diversity of intestinal flora would lead to the reduction of the host’s resistance to pathogens [37]. In our research, hMSCs treatment reversed the reduction of intestinal microflora in NASH induced by the MCD diet, but findings did not achieve statistical significance; findings suggested that hMSCs could increase the diversity of flora. In the current study, hMSCs treatment did not remarkably improve the diversity of intestinal microflora probably due to the rare number of specimens or the insufficient treatment cycle of hMSCs. Moreover, the diversity of intestinal flora was significantly different between the MCD and chow groups, and hMSCs treatment reduced the diversity of flora between the two groups. The above results showed that hMSCs could ameliorate NASH-related lesions and affect the intestinal flora.

The bacteria colonized in the intestine and the integrity of the intestinal mucosa together form the mucosal barrier of the intestine, which is a defense against pathogens [1]. The pathogenesis of many disorders could occur with the alteration of the intestinal microflora and destruction of homeostasis [38]. On the surface of protective mucus layer, mucins are abundant. Mucins contains glycoproteins and can be directly decomposed by some bacteria as a source of carbon and nitrogen for themselves and bacteria that are unable to degrade the outer mucin [39]. *Akkermansiaceae* is one of the bacteria that could decompose and utilize mucins [40]. Some studies have shown that *Akkermansiaceae*, which is a member of *Verrucomicrobia*, could help resist obesity, improve insulin resistance, and inhibit inflammation [41, 42]. In our study, *Akkermansiaceae* increased obviously in the MCD group compared with the chow group, which was inconsistent with previous research [43]. The reason for this difference might be that MCD diet disrupted barrier homeostasis and led to the reactive proliferation of *Akkermansiaceae*. However, after the hMSCs treatment, *Akkermansiaceae* decreased, but this finding did not reach statistical significance possibly due to the above mentioned limitations of our study.

*Erysipelotrichaceae* is an opportunistic pathogen that could translocate and secrete endotoxins, affect lipid metabolism, and aggravate inflammation [44]. In our study, we found that the quantity of *Erysipelotrichaceae* significantly increased in the MCD group compared with the chow group consistent with the previous findings [45] and decreased remarkably after hMSCs treatment. *Muribaculaceae* could produce short-chain fatty acid (SCFA), which could regulate intestinal pH value, inhibit the proliferation of harmful bacteria, and protect the homeostasis of intestinal environment [44]. In our study, *Muribaculaceae* was significantly reduced in the MCD group compared with the chow group, and hMSCs treatment slightly increased *Muribaculaceae*. At the genus level, *Desulfovibrio* was a potential pathogen that produced LPS and damaged the integrity of the intestinal mucosa [46], which was significantly increased in the MCD group compared with the chow group, and was decreased remarkably in the hMSCs treatment group. *Odoribacter* is a potential marker of liver cancer [47]. Its quantity was elevated in inflammation-related colon cancer [48]. In our study, *Odoribacter* increased after MCD diet and decreased to normal after hMSCs therapy. *P. distasonis* protects intestinal integrity and reduces intestinal tumors and inflammatory mediators [49]; it could also be in the gut to generate succinate and secondary bile acids and fight obesity and obesity-related metabolic syndrome [50]. hMSCs treatment could increase *P. distasonis* significantly compared with the MCD group. Furthermore, as shown in LEfSe analysis, MCD diet decreased the quantity of probiotics such as *lactobacillusaceae*, *Rikenellaceae*, *Alistipes*, and *Muribaculaceae* and increased the quantity of opportunistic pathogen (*Akkermansiaceae* and *Erysipelotrichaceae*) and LPS-producing bacteria (*Faecalibaculum*) compared with the chow group. hMSCs treatment reversed the change of flora and increased Clostridia, which could reduce lipid deposition by decreasing the uptake of long-chain fatty acids. Meanwhile, Clostridia is also negatively correlated with TNF-a and IL-6 in the liver [51]. Therefore, these data indicated that hMSCs treatment could play an important role in regulating intestinal flora of mice fed with MCD diet to induce NASH.

To link the microbial community structure and metabolic functions, we studied the KEGG metabolic pathways. In our study, compared with *Erysipelotrichaeae* and *Desulfovibrio*, *Lactobacillusaceae* and *Clostridiales* were more abundant in the lipid metabolism pathway, which indicated that *Lactobacillusaceae* and *Clostridiales* were more prone to lipid metabolism. Differences in the metabolic pathways were found among microbial communities in the three groups. Many metabolic pathways were downregulated in the MCD group compared with the chow group, such as lipid, carbohydrate, and energy metabolism. The pathways of drug resistance and membrane transport were upregulated in the MCD group. The hMSCs treatment improved the metabolic pathway disorder induced by the MCD diet. Although our study found an association between altered metabolic pathways and NASH, the causative contribution of the metabolic pathways to NASH progression was not sufficiently elaborated. Therefore, more research needs to be done on metabolic pathways in the later stage.

Most bacteria in the gastrointestinal tract were considered commensals, but they could also inhibit other bacteria. *Desulfovibrionale*, for example, could produce LPS and further aggravate inflammation [46], and it was positively correlated with *Odoribacter*, which is a potential marker of liver cancer and is associated with inflammation-related colon cancer [48]. *Desulfovibrionales* was also negatively correlated with *Anaerotruncus*, which colonizes the outer mucus layer of the colon and is a butyrate-producing taxa. Butyrate plays an important role in maintaining the health of bacteria, because it is the main energy source of the colonic mucosa; it could adjust the gene expression of the host cell, inflammation, and apoptosis of cells [52]. Excessive Proliferation of *Akkermansiaceae* would destroy the integrity of the gut, and it showed negative correlation with *Muribaculum*, which protects the intestinal mucosa and stabilized intestinal homeostasis. Meanwhile, *Akkermansiaceae* was negatively correlated with *Turicibacter*, which is an opportunistic pathogen that produces inflammatory mediators [9]. Moreover, *Muribaculum* was negatively correlated with *Faecalibaculum*, which could increase liver weight, promote the expression of LPS and inflammatory factors, and increase the liquification fraction of the liver [44]. Flora and metabolic pathways constituted a feedback loop in the microbiota ecosystem, which substantially amplified the effects of the bacteria on the host.

Our study demonstrated that hMSCs could reverse the disorder of NASH mice flora induced by MCD diet, which might link the liver microenvironment with the intestinal microecology and provide a new mechanism for hMSCs to improve NASH-related lesions. However, our study had some limitations. First, although the pathological changes of our NASH model, which was characterized by hepatic steatosis and inflammatory infiltration, resembled those of human NASH, our model lacked the obesity and insulin resistance characteristics of NASH patients. So, further clinical studies are needed. Second, we found that hMSCs treatment could improve NASH-related lesions and intestinal flora disorder induced by MCD diet, but some of the changes attained were not statistically significant. In our future study, we will further optimize the experimental conditions and obtain more valuable experimental results.

## MATERIALS AND METHODS

### Animals and diet

Six to eight-week-old male C57BL/6 mice were purchased from Branch of Beijing Vital River Laboratory Animal Technology Co., Ltd. (Beijing, China) and maintained under a 12 h light/12 h dark cycle with free access to food and water. The mice were acclimatized for 1 week with free access to a standard chow diet and water and randomly divided into two groups, as follows: (i) chow, controls fed with a regular diet, n=5 and (ii) MCD group, mice fed with MCD diet, n = 10. After 10 weeks, MCD mice were randomized into two groups again, as follows: (ii) MCD, untreated mice from the MCD group, n = 5; and (iii) MCD + hMSCs, mice fed with the MCD diet supplemented with an intravenous injection of 1×10^6^ hMSCs administered at 10 weeks of the 12-week experiment, n = 5. At 12 weeks, all mice were euthanized, and liver tissues were harvested.

### Isolation and culture of umbilical cord-derived hMSCs

The study involving human material was approved by the Ethics Committee at the Research Center for Obstetrics, Gynecology, and Perinatology. Umbilical cords were collected after normal deliveries, and each mother signed an informed consent.

Subsequently, we isolated and characterized the hMSCs extracted from the umbilical cords according to procedures approved by the Ethics Committee at the Chinese Academy of Medical Sciences and Peking Union Medical College. Some minor modifications have been made on the previously described procedure [53].

Each cord was washed out with D-Hanks solution, the cord vein was catheterized and washed internally by phosphate-buffered saline (PBS) with 1% penicillin and streptomycin solution. We removed the blood vessels and cut umbilical cords into small pieces with scissors. Then, we incubated the pieces with 0.2% collagenase type I (ITS; Gibco, USA) for 2.5 h at 37 °C and 220 r/min. We plated the cells on a cell culture flask at a density of 2 × 10^6^ cells/ml. The cells were cultured in a DMEM/F-12 medium (Gibco Life Technologies, Paisley, UK) supplemented with 2% fetal bovine serum (FBS; Gibco, USA) and maintained at 37 °C and 5% CO_2_. We used the cells in our experiments after identifying the phenotype of hMSCs via flow cytometry.

### Immunophenotyping of MSC-like cells by flow cytometry

For flow cytometry analysis, expanded cells were assessed using the BD Stemflow™ hMSCs Analysis Kit (BD Biosciences) for the presence of cell surface antigens CD73, CD90, and CD105 and the absence of CD34, CD45, CD11b, CD19, and HLA-DR, according to the methods in a previous report [54]. The cells were detached and washed twice with PBS and incubated with antibodies for 30 min at room temperature. Isotype controls were used for each antibody to detect the nonspecific background signal as negative controls. Stained cells were analyzed using BD FACSCanto II. We also used BD FACSDiva software for analysis and used the same setup for all tested populations. Data were analyzed using Flow Jo 7.6 software (BD Biosciences, USA).

### Histology of livers

For the liver tissue sections, 4% paraformaldehyde (PFA) was used as a fixative. The sections were then embedded in paraffin. Subsequently, we cut the liver tissue into 2 μm-thick sections for staining with hematoxylin and eosin (HE) and Sirius Red. Then, we evaluated the hepatic steatosis, inflammation, ballooning, and liver fibrosis using microscope of OLYMPUS BX53 according to the NAFLD activity score (NAS) system [27]. Each image took 10 views, which basically contained all parts of the image. Finally, we used Graphpad Prism 8.0 software for analysis.

### Quantitative real-time PCR

We used the total RNA (1 μg) extracted from the liver tissues by the TRIzol-glycogen method to generate cDNA according to the manufacturer’s instructions of PrimeScript^TM^RT reagent Kit with gDNA Eraser (Perfect Real Time). qPCR was performed using TB Green Premix Ex Taq^TM^ (Tli RNaseH Plus) and routinely performed on StepOnePlus Real-Time PCR System. The relative expression levels of mRNAs were normalized to that of β-actin mRNA. The relative quantification of gene expression was based on the comparative CT method, in which the number of targets was given by 2^–ΔΔCt^. The designed RT-qPCR primers were shown in Table 1.

**Table 1:** Primers designed for RT- qPCR.

	**Forward primer sequences**	**Reverse primer sequences**
a-SMA	5’ -GTCCCAGACATCAGGGAGTAA-3’	5’ - TCGGATACTTCAGCGTCAGGA -3’
CTGF	5’- GGGCCTCTTCTGCGATTTC -3’	5’- ATCCAGGCAAGTGCATTGGTA -3’
CXCL2	5’- CCAACCACCAGGCTACAGG -3’	5’- GCGTCACACTCAAGCTCTG -3’
TNF-α	5’- CCCTCACACTCAGATCATCTTCT -3’	5’- GCTACGACGTGGGCTACAG -3’
B-actin	5’- GAAGATCAAGATCATTGCTCCT -3’	5’- TGGAAGGTGGACAGTGAG -3’

### Gut microbiota analysis

When the mice were killed at 12 weeks, feces were harvested from the colon. Three or four feces were collected from each mouse. Subsequently, these feces were separately cryopreserved until further analyses.

After extracting the total DNA of the sample, according to the 16S full-length primers 27F/1492R, a specific primer with Barcode was synthesized. Subsequently, after amplification by PCR, purification, quantification, and homogenization of the 16S rRNA genes were performed, and we used the product to form a sequencing library (SMRT Bell). The constructed library was tested first, and sequenced by PacBio Sequal. The original data was spliced by FLASH (version 1.2.11). The quality of spliced sequence was filtered by Trimmatotic (version 0.33), and the chimera was removed by UCHIME (version 8.1) to obtain high quality tag sequences. The sequences were clustered at 97% similarity level (USEARCH, version 10.0), and OTU was filtered by 0.005% of all sequences.

Subsequently, we used the packages of R software to measure the a-diversity indexes (Chao1) and b-diversity (PCoA) among the three groups. Linear discriminant analysis (LDA) Effect Size algorithm (LEfSe) were used to identify differences in the abundance of OTUs among the three groups with log 10 LDA scores >4.0. We used PICRUST software to predict metagenomic functional information based on the OTU table. We used STAMP software to determine the statistical differences of KEGG pathways among the three groups. Furthermore, a network diagram of bacterial interaction was constructed by Spearman rank correlation analysis.

### Statistical analysis

Results were presented as mean ± SEM. Statistical analyses were performed using GraphPad Prism version 8.0 (California, UK). The Student's-test was used for the statistical analysis of all independent experiments. Comparisons of parameters for three groups were made by one-way analysis of variance (ANOVA) followed by Tukey's test. A value of P < 0.05 was considered statistically significant.

### Ethical approval

All procedures performed in studies involving animal experiments were in accordance with the ethical standards of the Animal Ethics Committee of Zhengzhou University and with the National Institutes of Health Guide for the care and use of Laboratory animals (NIH Publications No. 8023, revised 1978).
